# A primer on meta-awareness of mind wandering in schizotypy and schizophrenia

**DOI:** 10.1007/s00426-025-02172-7

**Published:** 2025-10-04

**Authors:** Anchal Garg, Bruce D. Watt, Mohamad El Haj, Ahmed A. Moustafa

**Affiliations:** 1 School of Psychology, Faculty of Society and Design, Bond University, Robina, Gold Coast, QLD 4226 Australia; 2 Laboratoire de Psychologie des Pays de la Loire (LPPL EA 4638), Nantes Université, Univ Angers, Nantes, France; 3 Department of Human Anatomy and Physiology, Faculty of Health Sciences, University of Johannesburg, P.O. Box 524, Johannesburg, South Africa

**Keywords:** Metacognition, Attention, Spontaneous cognition, Schizotypal personality, Psychosis, Negative schizotypy, Disorganised schizotypy, Daydreaming

## Abstract

During everyday life, our attention may drift away from immediate perceptual inputs to consider alternatives unrelated to the task at hand, which is known as mind wandering. At times, mind wandering may be accompanied by awareness of one’s attention having deviated from the ongoing task, often referred to as meta-awareness. However, mind wandering can also occur without individuals immediately noticing that their attention is off task. Importantly, little is known about mind wandering and meta-awareness, including their underlying content in schizotypy and schizophrenia. Here, in this narrative review, we synthesise the existing literature on mind wandering across the schizotypy-schizophrenia continuum. In doing so, we present the main proposal that there would be lower levels of meta-awareness of mind wandering in patients with schizophrenia as compared to healthy controls. We expect that meta-awareness of mind wandering would be negatively associated with schizophrenia symptomatology and multidimensional schizotypy traits. We also recommend examining mind wandering content in patients with schizophrenia spectrum disorders, including links between multidimensional schizotypy and the contents of meta-awareness of mind wandering. Ultimately, we hope that our proposal will be empirically tested in future research and will provide a foundation to understand the diversity of mind wandering features in clinical populations as well as insights into potential new treatment avenues.

## Mind wandering

An intriguing aspect of human cognition is the tendency to withdraw attention from our immediate perceptual inputs (i.e., perceptual decoupling), and focus on information unrelated to the current situation and/or task at hand (Schooler et al., 2011; Stawarczyk et al., 2011). This phenomenon of so-called mind wandering often encompasses memories of the past, planning, and anticipation of future events, or socially relevant forms of thinking (e.g., Smallwood & Andrews-Hanna, 2013). Mind wandering appears to be a ubiquitous occurrence, and has been suggested to account for 20–50% of our waking cognition (e.g., Kawashima et al., 2023; Killingsworth & Gilbert, 2010; Song & Wang, 2012). These estimates of mind wandering in daily life suggest that this phenomenon may confer some adaptive benefits. Mounting evidence indicates that mind wandering is indeed associated with various benefits, including decision-making, planning, creative thinking, and goal-directed thinking (e.g., Fox & Christoff, 2014; Mooneyham & Schooler, 2013). At the same time, mind wandering is associated with significant costs, such as disruptions in learning (e.g., comprehension deficits), poor psychological well-being, and risky driving behaviours (e.g., Mooneyham & Schooler, 2013; Yanko & Spalek, 2014). Overall, different expressions of mind wandering can be useful in understanding the adaptive and maladaptive consequences of inner mentation.

It is important to mention that mind wandering is considered a multidimensional construct with numerous definitions in the literature, such as off-task thinking, unintentional thoughts, stimulus-independent thoughts, self-generated thoughts, and spontaneous cognition (e.g., Gonçalves et al., 2020; Seli et al., 2018). Therefore, to avoid lumping together different varieties of mind wandering (cf. Christoff et al., 2018), in this review, we operationalise mind wandering as a state of perceptual decoupling from the current external environment and redirection of attentional resources towards processing and maintenance of internally generated thoughts (i.e., content-based criterion; Schooler et al., 2011). This operationalisation is commonly used in schizophrenia and schizotypy research and enables the differentiation of mind wandering from other conscious experiences, such as on-task reports, task-related interferences, and external distractions (Stawarczyk et al., 2011; Van den Driessche et al., 2025).

### Objectives of the Review

Understanding how patients with schizophrenia monitor their own thought processes has significant implications for psychopathology. Therefore, in this paper, we aim to synthesise the existing mind wandering literature across the schizotypy-schizophrenia continuum and propose that the alterations in meta-awareness of mind wandering are linked to schizotypy traits and schizophrenia symptomatology. We also provide methodological recommendations to test our proposal. Finally, we emphasise the importance of studying mind wandering contents in patients with schizophrenia, including an investigation of links between schizotypy and underlying content of meta-awareness of mind wandering. We believe that our proposal provides unique insights into current conceptualisations of internal mentation, including implications for treatment modalities requiring awareness of thought content and processes. For instance, enhancing meta-awareness for patients with schizophrenia may reflect an initial treatment target to facilitate successful outcomes (e.g., recovery). We think that narrative review is the most suitable approach as the aims are to provide detailed interpretation of findings on mind wandering across schizotypy-schizophrenia continuum, along with advancing the field through our novel proposal, which reflects an intersection between clinical psychology and cognitive psychology (Greenhalgh et al., 2018).

### The contents of mind wandering

A prominent theme in the literature indicates that the contents of mind wandering episodes may be a critical factor in determining its underlying costs and benefits. The *content regulation hypothesis* (Smallwood & Andrews-Hanna, 2013) posits that mind wandering can benefit individuals who have the ability to regulate the contents of such inner mentation by focusing on positive or productive themes (Andrews-Hanna et al., 2013). In contrast, individuals who lack this capacity to regulate their inner mentation may experience maladaptive psychological consequences (Andrews-Hanna et al., 2013; Smallwood & Andrews-Hanna, 2013). Consistent with this hypothesis, Andrews-Hanna et al. (2013) found that individuals who reported more personally significant and negative thoughts scored higher on constructs associated with depression/negative affect (i.e., poor psychological well-being). Similarly, individuals who generated more abstract and less specific thoughts scored higher on constructs linked with rumination (Andrews-Hanna et al., 2013). On the other hand, individuals who characterised their thoughts as more positive, less personally significant, and more specific and concrete scored higher on dispositional mindfulness (i.e., improved well-being; Andrews-Hanna et al., 2013). These findings underscore the importance of understanding how the content of people’s inner thoughts potentially determines the associated costs and benefits of mind wandering.

Mind wandering can often, but not always, be accompanied by the realisation that one’s thoughts have wandered away from the situation and/or task at hand, often referred to as *meta-awareness* (Schooler, 2002; Schooler et al., 2011). In fact, meta-awareness of the wandering mind frequently occurs throughout our day-to-day experiences depending on current environmental, contextual, and motivational constraints (e.g., Smallwood & Andrews-Hanna, 2013; Zedelius et al., 2015). For instance, when reading an unengaging article, a student may start thinking about their upcoming vacation, later “catching themselves” mindlessly looking at the article, before resuming reading. The precise interplay between meta-awareness and mind wandering remains unclear; however, mounting evidence suggests their interaction during a variety of cognitive experiences, including creative thinking, mindfulness meditation, and lucid dreaming (e.g., Fox & Christoff, 2014).

Empirical evidence suggests that meta-awareness of mind wandering may have clinical implications across a range of psychopathological/sub-clinical conditions, including Attention-Deficit/Hyperactivity Disorder (ADHD), depression, and Alzheimer’s disease (e.g., Deng et al., 2014; Franklin et al., 2017). At the same time, despite the extensive literature suggesting the prevalence of metacognitive deficits across the schizotypy continuum, including schizophrenia spectrum conditions (e.g., Garg & Singh, 2016; Lysaker et al., 2013), to date, no studies have examined meta-awareness of mind wandering in these populations.

## Meta-Awareness

Meta-awareness can be defined as “the ability to reflect upon the content of one’s own mental state” (Smallwood et al., 2007, p. 527). Meta-consciousness (or meta-awareness) is considered a monitoring system (Seli et al., 2017), that is engaged intermittently to notice contents of the basic consciousness, such as how happy do I feel at the moment? (Schooler, 2002; Schooler et al., 2015), and to assess whether current mental contents are consistent with the goals of the individual (Seli et al., 2017; Smallwood, 2013). Meta-consciousness allows one to become explicitly aware of their mind wandering episodes (Schooler, 2002; Schooler et al., 2011). Thus, it has been suggested that, “the resulting meta-consciousness involves an explicit re-representation of basic consciousness in which one interprets, describes, or otherwise characterizes the state of one’s mind” (Schooler, 2002, p. 340). Interestingly, one can be meta-aware of some aspects of their experience while being oblivious to other aspects (Schooler, 2002). For instance, during a long drive, an individual is aware that they have been thinking of their partner, but is not explicitly aware of the associated sadness. Alternatively, meta-awareness can co-occur (or nearly so) with the experience to which it corresponds or can be imposed retrospectively when reflecting on prior experiences (Schooler, 2002). Thus, meta-consciousness can be directed towards any aspect of experiences and explicitly appraises the contents of consciousness.

## Meta-Awareness of Mind wandering

**Fig. 1:**
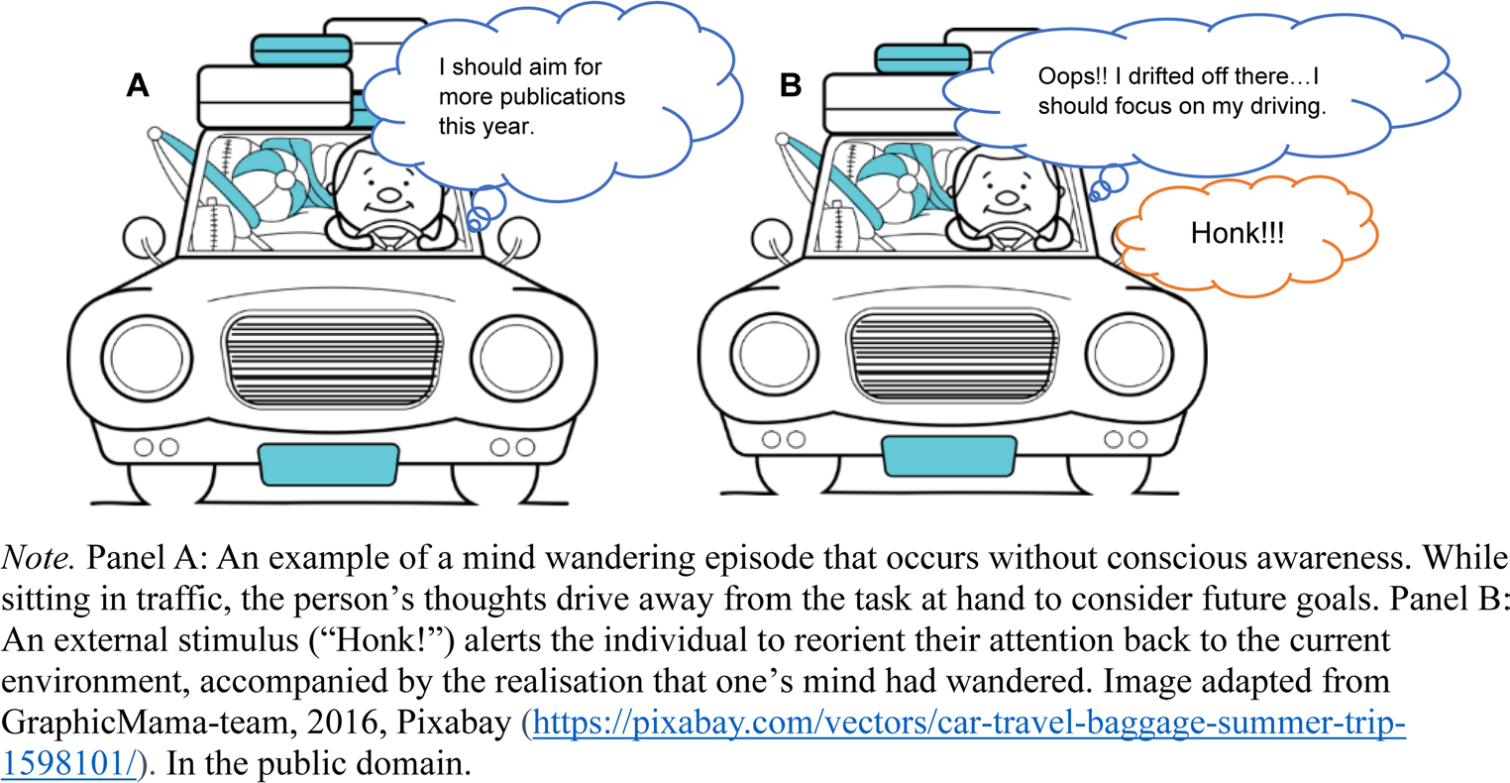
Mind wandering without awareness in daily life

Research on the occurrence and consequences of mind wandering is often complemented by the meta-awareness dimension; an investigation about one’s ability to notice their mind wandering episodes (Schooler et al., 2011). Two states of mind wandering have been proposed to vary in terms of meta-awareness: *tuning out* and *zoning out*, which refer to mind wandering with and without meta-awareness, respectively (Smallwood et al., 2007, 2008). In other words, during tune outs/aware mind wandering, one is fully cognizant of the fact that their mind has drifted away from the current situation and/or task at hand (Jackson & Balota, 2012). For example, while reading a book chapter, a student can be aware of the holiday ideas coming to their mind, but for some reason, they continue to read. On the other hand, during zone outs/unaware mind wandering, one does not recognise that their mind has drifted away until they catch themselves or are probed directly (Jackson & Balota, 2012). In other words, “when people zone out, they are experientially conscious of whatever topic has grabbed their attention, while at the same time lacking metaconsciousness of the fact that they are zoning out” (Schooler et al., 2004, p. 203). For example, while waiting at a set of traffic lights, a researcher might drift off to consider their academic goals for the current year when the sudden blare of a car horn alerts them that the lights have, in fact, changed to green (Fig. 1). This is also accompanied by a sudden realisation that they had been mind wandering (i.e., “Oops!! I drifted off there”), and they should focus on driving again (Fig. 1B). The driving example highlights two key points: (a) during zone outs, one does not explicitly realise that they are mind wandering, but at the same time, can phenomenally experience the content of such episodes (Chin et al., 2012; Fabry, 2018), and (b) the occurrence of zone outs can be established retrospectively (if at all; Smallwood & Schooler, 2015; Takarangi et al., 2015).

Meta-awareness and mind wandering dynamics play an important role in our day-to-day experiences. In relation to Fig. 1, the interplay is *corrective* in nature as meta-awareness may have identified the occurrence of mind wandering episode and re-directed the researcher’s attention back to the driving task (Fox & Christoff, 2014; Schooler et al., 2011). Meta-awareness and mind wandering may further interact in a *positive and facilitative* fashion across several cognitive experiences, including creative thinking, mindfulness meditation, yoga-based practices (e.g., open monitoring techniques), and lucid dreaming (e.g., Fox & Christoff, 2014; Windt & Voss, 2018). An example of this interaction could be in creative thinking, when mind wandering might play a role in the generation of new ideas, whereas meta-awareness may contribute towards being aware of these new self-generated ideas (Fox & Christoff, 2014). One possible outcome of this interaction can be coming up with novel conclusions or insights (e.g., aha! moments) that might not be achieved if one was not explicitly aware of the ideas generated through mind wandering (Fox & Christoff, 2014). Thus, mind wandering and meta-awareness interaction offers insights regarding how the mind toggles between externally and endogenously driven processes.

## Clinical implications of Mind wandering

The study of mind wandering is increasingly recognised as holding important clinical implications for several disorders, including ADHD, depression, social anxiety disorder, schizophrenia, and dementia (e.g., Madiouni et al., 2020; O’Callaghan et al., 2019). Among these conditions, schizophrenia is of considerable interest given hallmark changes in thought processes and contents. Patients often report feeling detached from external reality and can withdraw into their own private world (Parnas, 2011; Stanghellini & Ballerini, 2007). This internal cognitive retreat bears similarities to the perceptual decoupling and internally directed features of mind wandering, which also manifests in the core symptoms of schizophrenia, such as hallucinations, delusions, and impairment in reality processing (Chen et al., 2019). Schizotypy is also of considerable interest as “schizotypy is thought to represent the phenotypic manifestation of the underlying vulnerability for schizophrenia-spectrum psychopathology that is expressed across a broad range from subclinical expression to the prodrome to schizophrenia-spectrum personality disorders to full-blown psychosis” (Kwapil et al., 2018, p. 209). Previous studies demonstrate that mind wandering rates in non-clinical samples are positively correlated with schizotypy traits (e.g., strange perceptual experiences, persecution, and cognitive difficulties; Kane et al., 2016) and the total schizotypy score on the Schizotypal Personality Questionnaire Brief (Yamaoka & Yukawa, 2020). In other words, examining the links between mind wandering and schizotypy traits in a non-clinical sample could provide unique insights into the internal mentation of individuals with schizophrenia spectrum psychopathology, without the confounds associated with these clinical conditions (e.g., medications and hospitalisations; Kwapil & Barrantes-Vidal, 2015).

### A schizotypy-schizophrenia continuum

Recent theoretical frameworks have discussed mind wandering as a dimensional construct that unfolds depending on current task demands, environmental constraints, and the individual’s physiological and psychological state (Christoff et al., 2016; O’Callaghan et al., 2015). Understanding variations in mind wandering along a putative schizotypy-schizophrenia continuum can potentially accommodate shifts in adaptive and maladaptive forms of mind wandering observed in clinical populations. Here, we consider the schizophrenia-schizotypy continuum mainly through the lens of the Fully Dimensional Model (FDM), which is based on dimensional models of personality and psychopathology (Rawlings et al., 2008). The FDM defines schizotypy as a cluster of enduring personality traits, characterised by distinct cognitive styles and perceptual experiences, arising from a combination of genetic and environmental variations that are normally distributed in the general population (Claridge, 1985; Green et al., 2008). The FDM posits that the latent structure of schizotypy lies on a continuum that applies to all members of the general population (Nelson et al., 2013). Specifically, this continuum ranges from low levels of schizotypy and psychological health to extremely high levels of schizotypy and potential dysfunction in the form of schizophrenia spectrum disorders (Mohr & Claridge, 2015; Nelson et al., 2013). The FDM also posits a clear demarcation between health and illness along the schizotypy-schizophrenia continuum, where signs of impaired functioning are used to denote disorders (Claridge & Beech, 1995; Grant et al., 2018). Nelson et al. (2013) noted that high levels of schizotypy combined with other aetiological risk factors (e.g., negative life experiences) could predispose an individual to a greater risk of developing schizophrenia and related disorders. Overall, the FDM operationalises that schizotypy has dual properties; it represents adaptive variation in personality, yet has the potential for psychopathological manifestations (Garg et al., 2025; Grant et al., 2018; Rawlings et al., 2008).

### Features of schizotypy versus schizophrenia

Schizotypy is a multidimensional construct consisting of positive, negative, and disorganised dimensions (Kwapil & Barrantes-Vidal, 2015). Positive schizotypy refers to excessive or distorted functioning of normal processes, including disruptions in thought content (e.g., magical ideation), perceptual disturbances, and suspiciousness/paranoia (Fonseca-Pedrero et al., 2007; Kwapil et al., 2018). Negative schizotypy refers to diminution or deficit in one’s normal behaviour (Fonseca-Pedrero et al., 2007), such as alogia and social disinterest (Kwapil et al., 2018). Disorganised schizotypy refers to disturbances in organisation and expression of thoughts, speech, and behaviour, which could range from mild disturbances to formal thought disorders and extremely disorganised behaviours (Kwapil et al., 2018). By contrast, schizophrenia is a clinical syndrome characterised by abnormalities in cognition, emotion, perception, and other aspects of behaviour (Sadock & Sadock, 2011). A diagnosis of schizophrenia involves the presence of two (or more) of the following symptoms: delusions, hallucinations, disorganised speech, grossly disorganised or catatonic behaviour, and negative symptoms (e.g., avolition; American Psychiatric Association, 2013). The diagnosis of schizophrenia also involves impairment in functioning in one or more major areas, such as work, interpersonal relationships, or self-care, and continuous signs of disturbance last for at least 6 months, including 1 month of active phase of symptoms (American Psychiatric Association, 2013).

## Mind wandering in schizophrenia and schizotypy: A critical angle

Altered thought processes are characteristic of schizophrenia, often in the form of excessive variability (e.g., flight of ideas), suggesting an increase in the diversity of mind wandering, whereas excessive stability of thoughts (e.g., rigid thinking) is indicative of a decrease in mind wandering (Christoff et al., 2016). Despite the clear implications for internal mentation, very few studies have explored how mind wandering is affected in the schizophrenia population (see Table 1). For instance, Shin et al. (2015) found an elevation of trait mind wandering on the Mind Wandering Frequency Scale in patients with schizophrenia relative to healthy controls, which was also positively correlated with positive symptoms measured by PANSS. Using the Sustained Attention to Response Task (SART) along with thought probes, Chen et al. (2019) operationalised mind wandering as stimulus-independent and task-unrelated thoughts (SITUT), and found that patients with schizophrenia reported a lower frequency of mind wandering as compared to healthy controls. No correlations were observed between the frequency of mind wandering and schizophrenia symptoms, which could be due to a small sample size in the study (Chen et al., 2019). Another study by Iglesias-Parro et al. (2020) used a naturalistic approach to explore mind wandering using experience sampling probes during the presentation of video clips in which auditory and visual information was synchronous or asynchronous. In both the synchrony and non-synchrony conditions, patients with schizophrenia showed a higher frequency of mind wandering as compared to healthy controls, yet no correlation was evident between mind wandering and schizophrenia symptomatology (Iglesias-Parro et al., 2020). It is worth noting that this study also included patients with schizoaffective and psychotic disorders, which limits our capacity to draw definitive conclusions regarding mind wandering in schizophrenia. Thus, the few studies to explore mind wandering in schizophrenia present a rather mixed picture. We suspect that these contradictory findings likely reflect differences in mind wandering assessments (self-report versus experimental task) as well as the way in which mind wandering categories were operationalised (e.g., task-relatedness and stimulus-dependency dimensions). Most crucially, to report mind wandering episodes on these measures accurately, individuals need to be aware of their mental states, which is often impaired in patients with schizophrenia (Garg & Singh, 2016; Lysaker et al., 2020).

**Table 1:** A summary of studies on mind wandering in schizophrenia

Study	Sample	Schizophrenia assessment	MW assessment	Key finding
Shin et al. (2015)	33 SZP 33 HC	PANSS	MWQ (IPI)	SZP scored higher on trait MW than HC. Trait MW positively correlated with positive symptoms, but did not correlate with negative symptoms.
Chen et al. (2019)	58 Chronic SZP 56 HC	PANSS	SART-TP (Chinese version)	SZP reported a lower frequency of MW as compared to HC. MW frequency did not correlate with positive and negative symptoms.
Iglesias-Parro et al. (2020)	22 SSD 23 HC	PANSS	Series of 36 video clips (6 original audio-visually synchronised clips and 30 clips were not audio-visually synchronised clips)- TP	SZP showed higher MW frequency than HC across synchrony and non-synchrony conditions. MW frequency (both in synchrony and non-synchrony conditions) did not correlate with positive and negative symptoms.

*Note*. Only key findings relevant to this paper are summarised in the table. SZP* =* patients with schizophrenia, HC* =* healthy controls, PANSS = Positive and Negative Syndrome Scale, MWQ (IPI) = Mind Wandering Questionnaire, a subscale of the Imaginal Processes Inventory, MW* =* mind wandering, SART = Sustained Attention to Response Task, TP = thought probes, SSD = schizophrenia spectrum disorders

We believe it is timely to explore disturbances in meta-awareness of mind wandering in schizophrenia. To our knowledge, no study to date has included a formal assessment of meta-awareness in mind wandering research on schizophrenia, which represents an important direction for future research in this field. On the other hand, empirical evidence suggests that there are deficits in *metacognitive self-reflectivity* in patients with schizophrenia (e.g., Vohs et al., 2014). Metacognitive self-reflectivity conceptually overlaps with the meta-awareness of mind wandering, and refers to the capacity to be aware of and link one’s thoughts and feelings and their antecedents, that is, an integrated description of one’s mental state (Lysaker et al., 2010; Semerari et al., 2003). It also includes the ability to question one’s thoughts and to differentiate fantasy from reality (Lysaker et al., 2010; Semerari et al., 2003). Popolo et al. (2017) and others have found that patients with schizophrenia often display poorer metacognitive self-reflectivity as compared to controls and/or other clinical groups (e.g., bipolar disorder and substance use disorders; Tas et al., 2014; Vohs et al., 2014). It is worth noting that the focus of metacognitive self-reflectivity is broad, as it encompasses taking note of thoughts and feelings, whereas in the context of this review, the focus of meta-awareness is on one type of cognitive experience, that is, mind wandering. Overall, the empirical evidence suggests that if awareness of internal mentation is a deficit (or impaired) in patients with schizophrenia, then they will also likely experience difficulty noticing their mind wandering episodes (Iglesias-Parro et al., 2020).

Constraining the focus on schizotypy, to the best of our knowledge, five studies have examined the relationship between mind wandering and schizotypy, as summarised in Table 2. Kane et al. (2016) conducted a large correlational study on mind wandering and schizotypy. Mind wandering was assessed across five different cognitive tasks along with thought probes. Results indicated that individuals with a higher frequency of mind wandering (i.e., task-unrelated thoughts [TUT]) during ongoing cognitive performance are likely to report more strange perceptual experiences and beliefs (positive schizotypy), more confusion and cognitive difficulties (disorganised schizotypy), and more suspiciousness and persecution (paranoid schizotypy) in their daily lives (Kane et al., 2016). Similarly, Kane et al. (2021) examined the relationship between positive schizotypy and mind wandering. Specifically, mind wandering was examined using two cognitive tasks along with thought probes and the Mind Wandering Questionnaire was administered to assess trait levels of mind wandering. No associations were found between positive schizotypy traits and mind wandering frequency (TUT) or trait propensity for mind wandering (Kane et al., 2021). Chen et al. (2022) compared mind wandering frequency (SITUT) between individuals with high and low schizotypy using the SART along with thought probes. This study did not find any difference in mind wandering frequency between the two schizotypy groups (Chen et al., 2022). Finally, two studies examined the links between trait mind wandering and schizotypy. Yamaoka and Yukawa (2020) examined trait mind wandering (measured using the Japanese version of the Mind-Wandering Questionnaire) and found a positive correlation with total schizotypy score. Similarly, Zhang et al. (2022) found that individuals with high schizotypy scored higher on trait mind wandering (measured using the Chinese version of the Mind Wandering Questionnaire, a subscale of the Imaginal Processes Inventory) as compared to those with low schizotypy.

**Table 2:** A summary of studies on mind wandering and schizotypy

Study	Sample	Schizotypy assessment	MW assessment	Key finding
Kane et al. (2016)	541 UG	WSS SPQ PARACHEK COGSLIPG COGDYSRG	Semantic SART-TP Number Stroop-TP Arrow Flanker-TP Letter Flanker-TP 2-Back-TP	MW (TUT) positively correlated with positive, disorganised, and paranoid schizotypy. No correlation was found between MW and negative schizotypy.
Yamaoka and Yukawa (2020)	865 US	SPQ-Brief (Japanese version)	MWQ (Japanese version) ^a^	A positive correlation was found between schizotypal personality (the SPQ total score) and trait MW.
Kane et al. (2021)	1067 US	MIS (Short form)	MWQ (IPI) ^a^ Semantic SART-TP Arrow Flanker-TP	No correlations were observed between positive schizotypy and MW rates (TUT) measured on the Semantic SART and the Arrow Flanker task. No correlation was observed between trait MW measured via the MWQ (IPI) and positive schizotypy.
Chen et al. (2022)	55 participants (26 HS and 29 LS)	SPQ	SART-TP	No significant difference was observed between HS and LS groups regarding overall MW frequency. A lower proportion of future-oriented MW was observed in the HS group as compared to the LS group.
Zhang et al. (2022)	206 US (102 HS and 104 LS)	SPQ	MWQ (IPI; Chinese version) ^a^	The HS group scored higher on MW than the LS group.

*Note*. Only key findings relevant to this paper are summarised in the table. MW = mind wandering, UG* =* undergraduates, WSS = Wisconsin Schizotypy Scales, SPQ = Schizotypal Personality Questionnaire, PARACHEK* =* Paranoia Checklist, COGSLIPG = Cognitive Slippage Scale, COGDYSRG* =* Cognitive Dysregulation subscale of the Dimensional Assessment of Personality Pathology-Basic Questionnaire, SART* =* Sustained Attention to Response Task, TP* =* thought probes, TUT* =* task-unrelated thoughts, US = university students, MWQ = Mind-Wandering Questionnaire, MIS* =* Magical Ideation Scale, MWQ (IPI) = Mind Wandering Questionnaire, a subscale of the Imaginal Processes Inventory, HS = high schizotypal, LS* =* low schizotypal

^a^ It is worth noting that these studies used different MW measures. Specifically, Yamaoka and Yukawa (2020) assessed MW using the Japanese version of the Mind-Wandering Questionnaire (Kajimura & Nomura, 2016; Mrazek et al., 2013). On the other hand, Zhang et al. (2022) used the Chinese version of the Mind Wandering Questionnaire (Song & Tang, 2012), which is a subscale of the Imaginal Processes Inventory (Singer & Antrobus, 1963,1970), whereas Kane et al. (2021) used the English version of the Mind Wandering Questionnaire (Singer & Antrobus, 1970).

Considering these studies through a critical lens, with the exception of Kane et al. (2016), most studies treated schizotypy as a homogenous construct through the computation of a total schizotypy score. This failure to operationalise schizotypy as a multidimensional construct is not recommended as it can obscure the nature of associations with different dimensions of schizotypy (e.g., positive schizotypy), along which individuals are likely to vary (Kwapil & Barrantes-Vidal, 2015). Despite the prevalence of metacognitive deficits across the schizotypy continuum (Lehmann & Ettinger, 2023), to our knowledge, no study has examined the links of meta-awareness of mind wandering with multidimensional schizotypy suggesting a potential confound in these studies. In line with the literature on metacognitive self-reflectivity suggesting links with multidimensional schizotypy (Rabin et al., 2014), we believe that awareness of inner events is likely to be a negative correlate of multidimensional schizotypy. As a result, one can also expect a link between meta-awareness of mind wandering and multidimensional schizotypy.

In the context of phenomenology, to the best of our knowledge, only two studies have examined the links between schizotypy and mind wandering content (Chen et al., 2022; Welhaf et al., 2020). Specifically, Welhaf et al. (2020) examined the links by analysing a rich dataset from a previous study (i.e., Kane et al., 2016) and found positive associations of disorganised and positive schizotypy with both fantastical thoughts (daydreaming) and personal worry related content during mind wandering. A positive correlation was also observed between disorganised schizotypy and a greater variation in thought contents/topics (i.e., more TUT switches; Welhaf et al., 2020). No associations were observed between mind wandering content and negative schizotypy. Welhaf et al. (2020) did not analyse the data related to paranoid schizotypy. Chen et al. (2022) found a lower proportion of future-oriented mind wandering in individuals with high schizotypal traits than those with low schizotypal traits, suggesting that future-directed thinking might be compromised in individuals with high schizotypy traits (as summarised in Table 2). Methodological limitations are evident for this line of research. Chen et al. (2022), for example, did not examine whether future-oriented mind wandering is differentially linked with positive, negative, or disorganised schizotypy, as the focus was only on the SPQ total score. Further, such approach contrasts mind wandering research that has focussed on different subtypes of mind wandering. In the context of schizophrenia and related conditions, previous studies have not examined mind wandering content and its links with the schizophrenia symptomatology. Overall, previous mind wandering studies across schizotypy-schizophrenia continuum have not considered the meta-awareness dimension in their examination, which represents an important direction for future research in this field.

## Meta-awareness of mind wandering in schizophrenia and schizotypy: A proposal

In line with the extensive literature suggesting lower levels of metacognitive self-reflectivity in patients with schizophrenia (Vohs et al., 2014), we propose that there would be lower levels of aware mind wandering in patients with schizophrenia as compared to healthy controls. This hypothesis aligns with empirical evidence suggesting that impaired awareness of inner mentation in patients with schizophrenia may result in difficulty in taking note of their mind wandering episodes (Iglesias-Parro et al., 2020). In regard to schizophrenia symptomatology, mounting evidence suggests that lower levels of metacognitive self-reflectivity is associated with positive (Lysaker et al., 2005), negative, and disorganised (García-Mieres et al., 2020) symptoms, respectively. Therefore, we propose that meta-awareness of mind wandering would be negatively associated with positive, negative, and disorganised schizophrenia symptoms. The speculative hypothesis is that deficits in explicit awareness of inner experiences that is, mind wandering, is likely to contribute to detachment from the external world, resulting in bizarre interpretations of reality (e.g., delusions; Shin et al., 2015), cognitive disturbances (e.g., formal thought disorders), or diminished experiences or goal directed behaviours (e.g., avolition).

We constrain our focus on theories of consciousness, including the Higher-Order Thought, Global Neuronal Workspace, and Predictive Processing Theory. Among these theories, the Higher-Order Thought (HOT) theory is of considerable interest because of its direct emphasis on explicit awareness of mental states (Stefanelli, 2023), as compared to other theories, such as the Global Neuronal Workspace theory, which focuses on conscious and non-conscious states, but not meta-conscious state (Dehaene & Changeux, 2011). The HOT theory postulates that being conscious of our inner experiences requires a higher-order thought like representation (i.e., meta-representation; Lau & Rosenthal, 2011). Specifically, meta-representation refers to the capacity to represent and integrate one’s cognition and affect, which is believed to be impaired in patients with schizophrenia (Stefanelli, 2023). Such impairments lead to difficulties in making sense of their own and others’ experiences, contributing to schizophrenia symptoms, such as delusions and hallucinations (Stefanelli, 2023). In the context of mind wandering, impairments in meta-representation may reflect in reduced meta-awareness of mind wandering in patients with schizophrenia, making it difficult for them to recognise and monitor their mind wandering experiences. Finally, our proposal aligns with observations among patients with schizophrenia experiencing disturbances in the ability to reflect on their mental contents and lack of awareness about their cognitive functioning (cognitive insight) and symptomatology (clinical insight; Henriksen & Parnas, 2013).

The diversity of mind wandering can be studied in many ways, including temporal orientation, emotional valence, self-relevance, representational format (e.g., visual imagery), social orientation, and meta-awareness (e.g., Irish et al., 2019). On the other hand, there is no research examining the mind wandering content in schizophrenia population. Therefore, we recommend that future studies should examine phenomenology of mind wandering in this population and whether it is associated with the schizophrenia symptomatology. In line with the content-regulation hypothesis, we speculate that the cognitive-behavioural disorganisation in patients with schizophrenia (e.g., flight of ideas) might interfere with their ability to regulate the content of their inner mentation to productive themes, and therefore, they could experience maladaptive psychological consequences.

In the context of multidimensional schizotypy, Lynn et al. (2019) suggested that schizotypy is likely to be associated with deficits in meta-consciousness. The empirical evidence suggests correlations between metacognitive self-reflectivity and multidimensional schizotypy in a non-clinical sample (Rabin et al., 2014). Introspective abilities about one’s thoughts have been found to be negatively associated with multidimensional schizotypy (Lehmann & Ettinger, 2023), suggesting that schizotypy traits might interfere with one’s ability to note one’s thoughts. Similarly, we believe that individuals who lack motivation (negative schizotypy) and/or have cognitive disturbances (disorganised schizotypy) may struggle with taking a note of their mind wandering episodes. Therefore, we speculate that meta-awareness of mind wandering would be negatively associated with positive, negative, and disorganised schizotypy.

In line with the content-regulation hypothesis, we recommend examining whether there is any relationship between multidimensional schizotypy and the phenomenology of aware and unaware mind wandering; for instance, whether past-oriented unaware mind wandering episodes correlate with negative schizotypy. This line of investigation will address a research gap and will provide insights into whether aware and unaware mind wandering content is differentially linked with multidimensional schizotypy.

### Clinical implications

We believe that our proposal has clinical relevance as it could provide insights into whether meta-awareness mediates the links between mind wandering and schizophrenia symptoms, such as hallucinations. This speculation is in line with Franklin et al. (2017) who found that unaware mind wandering episodes mediated the relationship between the composite ADHD score and detrimental mind wandering. This suggests that increasing meta-awareness of mind wandering in individuals with ADHD could contribute towards improved functioning in daily life (Franklin et al., 2017), which might translate to the schizophrenia population if our research proposal obtains comparable results. Further, examining the links of meta-awareness of mind wandering with schizophrenia symptomatology/schizotypy traits could provide further support for the fully dimensional model.

Examining the links between mind wandering content and schizophrenia symptomatology could provide further support for the content-regulation hypothesis. This line of research could assist in understanding whether specific contents of internal mentation play a role in increasing or reducing the severity of schizophrenia symptomatology. Similarly, examining links between aware versus unaware mind wandering content and schizotypy could have potential clinical relevance. For example, common treatments for preventing the transition to psychosis, including Cognitive Behavioural Therapy, focus on thought patterns which requires meta-awareness (Hutton & Taylor, 2014; van der Gaag et al., 2012). If one is unable to identify their mind wandering episodes, then access to the underlying content will also be restricted during therapy, which might impact the treatment’s effectiveness. Enhancing meta-awareness for patients with schizophrenia may reflect an initial treatment target to facilitate gains with evidence-based interventions. At the same time, underlying benefits/drawbacks of aware and unaware mind wandering content for treatment effectiveness are also unknown, and therefore, could be examined in future studies.

### Methodological considerations

We recommend using tasks with low cognitive demands to assess mind wandering, meta-awareness, and the content in schizophrenia, such as the Shape Expectations Task (O’Callaghan et al., 2015). As noted by O’Callaghan et al. (2019), commonly used mind wandering assessments (e.g., SART) are usually dependent on dual-tasking and metacognitive capacities that are often compromised in neurodegenerative diseases or psychiatric conditions. As a result, such methodological approaches limit the extent to which reliable inferences can be drawn about mind wandering occurrences (O’Callaghan et al., 2019). Therefore, administering the Shape Expectations Task would enable examination of both occurrence and content of mind wandering/meta-awareness in the context of low cognitive demands, suitable for schizophrenia population. Another way to examine mind wandering could be using the ecological momentary assessment, which refers to the assessment of mind wandering in one’s natural environment using smartphones. In line with recommendations of Kawashima et al. (2023), we suggest that researchers take into account whether study parameters, such as measuring device (e.g., smartphone application), duration of the study, and frequency of thought probes, impact the mind wandering reports.

Neurocognitive studies have found differences in brain activity for aware versus unaware mind wandering states. For instance, Christoff et al. (2009) found that brain recruitments in both default network (e.g., posterior cingulate/precuneus) and executive network (e.g., dorsal anterior cingulate cortex) regions were stronger during zone outs (unaware mind wandering) as compared to tune outs (aware mind wandering). Moreover, no significant brain activations were observed during tune outs as compared to zone outs. Similarly, Boudewyn and Carter (2018) found a greater percentage of pre-probe alpha activity (often linked with the shifting of attention inwards and away from an external stimulus) in a scalp-recorded EEG just prior to zone outs as compared to tune outs. Therefore, to maximise measuring actual aware/unaware mind wandering experiences across schizophrenia-schizotypy continuum, we suggest combining Shape Expectations Task with techniques like EEG, eye-tracking, or fMRI (see also Zhang et al., 2024). We also recommend that studies should use the newly developed measure for schizotypy, the Multidimensional Schizotypy Scale (Kwapil et al., 2018). This scale was designed to examine the current conceptualisations of positive, negative, and disorganised schizotypy, and to overcome the conceptual and empirical limitations of the existing schizotypy measures (Kwapil et al., 2018).

## Conclusion

In this narrative review, we summarised the existing literature on mind wandering across the schizotypy-schizophrenia continuum and highlighted the lack of research on the meta-awareness dimension of mind wandering. Drawing on existing literature and theoretical frameworks, we propose that there would be lower levels of aware mind wandering in patients with schizophrenia as compared to healthy controls. Further, meta-awareness of mind wandering would be negatively correlated with schizophrenia symptomatology and multidimensional schizotypy. Our proposal paves the way for an important direction of research in mind wandering literature, including an investigation of its underlying phenomenology across schizophrenia-schizotypy continuum. Thus, this review contributes to fields of cognitive and clinical psychology and is relevant for researchers and clinicians focusing on cognition, metacognition, clinical/sub-clinical conditions, and treatment modalities of schizotypy and schizophrenia.

## Data Availability

No datasets were generated or analysed during the current study.

## Declarations

The manuscript does not contain clinical studies or patient data.
